# AD-DETR: A Real-Time Transformer with Multi-Scale Alignment and Spatial–Spectral Fusion for Crop Disease Detection

**DOI:** 10.3390/s26103206

**Published:** 2026-05-19

**Authors:** Bingyang Wang, Huibo Zhou, Zhi Wang, Ruolan Chen

**Affiliations:** School of Mathematical Sciences, Harbin Normal University, Harbin 150025, China

**Keywords:** crop disease detection, attention mechanism, RT-DETR, convolutional neural networks, deep learning

## Abstract

Agriculture faces significant challenges from crop diseases, which threaten global food security and cause substantial economic losses annually. While deep learning has advanced plant disease detection, existing models often struggle with generalization across heterogeneous environments and real-time deployment constraints, hindering their practical application in diverse agricultural settings. This paper proposes AD-DETR, an enhanced real-time detection transformer framework specifically designed for agricultural scenarios. The model incorporates three key innovations to address these issues. First, the Multi-Scale Align Network (MSANet) achieves adaptive feature alignment through an Adapt Fusion Align (AFA) block, effectively preserving disease detail information across varying scales. Second, the Spatial–Spectral Attentive Feature Fusion (SSAFF) module integrates frequency-domain processing with attention mechanisms, enhancing feature representation quality by combining spatial and spectral information. Third, the IPIoUv2 loss function improves bounding-box regression accuracy through an internal perception mechanism and scale-adaptive weighting. Comprehensive experiments demonstrate that AD-DETR achieves strong performance, with 90.2% mean average precision at IoU=0.5 on the Crop Disease dataset and 97.4% on the PlantDoc dataset. It maintains high efficiency with 16.4 million parameters, 47.2 GFLOPs computational complexity, and inference speeds of 230–242 frames per second. These results indicate that AD-DETR is robust to domain shift and suitable for resource-constrained applications, such as real-time monitoring on mobile and edge platforms.

## 1. Introduction

Agriculture acts as the mainstay of both economic prosperity and social progress on a national scale. Unfortunately, the rampant proliferation of crop diseases presents a formidable challenge to our food supply, the quality of agricultural goods, and the overall balance of agricultural environments [1]. The United Nations Food and Agriculture Organization (FAO) reports that between 20% and 40% of worldwide crop harvests are lost each year due to these destructive pests and diseases, translating into staggering economic damages worth hundreds of billions of dollars [2]. Conventional methods of diagnosing plant pathogens depend heavily on the seasoned judgment of agricultural specialists, an approach that suffers from steep labor expenses, sluggish processing times, and inconsistent evaluations—factors that fail to keep pace with the requirements of today’s data-driven, technologically advanced farming practices [3]. As a result, creating sophisticated and automated systems for identifying crop ailments has emerged as a critical focus area within the domain of agricultural innovation [4].

Early detection of plant diseases primarily relied on manual observation and laboratory analysis, which were inefficient and highly subjective. With technological advancements, traditional methods based on image processing techniques began to be applied in disease detection, such as using color and texture features for lesion segmentation. For instance, Loranger et al. [5] developed a Colour Analyzer tool based on HSV and Lab* color models for accurately measuring the area of leaf lesions. Bhujade et al. [6] proposed OptCFA method, which combines various filters such as GABF, AmPel, and E-SGF for image denoising, outperforming traditional filters. However, such conventional approaches struggle to adapt to different plant species and complex, variable field environments, exhibiting limited generalization capability.

Machine learning methods have further advanced the automation of plant disease detection. These approaches achieve disease identification by combining handcrafted features with classifiers. Sahu et al. [7] developed an HRF-MCSVM model that integrates spatial fuzzy C-means clustering and feature preprocessing to improve the accuracy of leaf disease detection. Selvaraj et al. [8] constructed a SqueezeNet model combined with the RCSO optimization algorithm, achieving high-sensitivity root disease classification under low-power constraints. Nevertheless, machine learning approaches’ performance heavily relies upon manual feature design quality, and they are often inadequate for handling large-scale, complex data.

During the previous decade, deep learning has become increasingly prevalent in computer vision. Convolutional neural networks (CNNs) have shown strong feature-extraction and image-recognition capabilities, creating new opportunities for intelligent crop disease diagnosis [9,10]. Scholarly inquiry in this domain has progressively moved beyond image classification to object detection and instance segmentation, which allow diseased areas to be localized and distinguished with greater accuracy. Gong and Zhang [11] developed an improved Faster R-CNN method for apple leaf disease detection, Arun et al. [12] proposed an enhanced tiny YOLO network for rice leaf disease detection, and Faisal et al. [13] used transfer learning with several CNN architectures for citrus plant disease classification. Zhang et al. [14] employed a ResNet-50-based network to evaluate tomato leaf disease severity. Related studies, including PlantVillage- and PlantDoc-based work, have also demonstrated that deep networks can learn discriminative disease features under both controlled and field-like settings [15,16,17,18].

Real-time object detection has been dominated by YOLO-series models, which prioritize inference speed but often struggle with complex multi-scale targets and domain shifts [19]. The introduction of real-time detection transformers, especially RT-DETR, marked a paradigm shift by combining the end-to-end detection capability of transformers with real-time performance [20]. RT-DETR uses a hybrid encoder with attention-based intra-scale feature interaction and cross-scale feature fusion to integrate multi-scale features. While RT-DETR achieves competitive accuracy and speed, its fixed-scale interaction mechanism lacks adaptability to the scale variations common in crop disease symptoms. Moreover, its reliance on spatial-domain processing overlooks the potential of frequency-domain information for capturing global texture patterns, a critical aspect for discriminating subtle disease lesions.

Multi-scale feature alignment is a core challenge in crop disease detection because disease symptoms exhibit high diversity across lesion sizes and developmental stages. Traditional CNNs extract hierarchical features, but they lack an explicit multi-scale alignment mechanism, resulting in semantic gaps and spatial misalignment between feature maps. Researchers have proposed methods to strengthen multi-scale representation. HRNet maintains high-resolution feature flows through parallel multi-resolution branches [21], while TridentNet uses multi-branch convolution kernels to process different scales [22]. Feature-pyramid networks have also been widely adopted for multi-scale object detection [23]. However, disease spots are often similar to healthy leaf vein textures, and cluttered backgrounds may cause fixed-scale networks to ignore small or low-contrast lesions.

Feature fusion technology integrates information at different levels or domains to improve representation quality. Spatial-domain methods have evolved from simple concatenation or element-wise addition to attention-based weighted fusion, such as channel attention and spatial attention that emphasize salient regions through adaptive weights [24]. Frequency-domain processing is also gaining attention in image analysis. Wavelet transform has been used to extract periodic patterns of leaf texture, while Fourier transform can improve global context modeling by enhancing edge and texture features [25,26]. In agricultural vision tasks, frequency-domain information can help capture disease distribution and texture patterns, but existing work is mostly limited to traditional signal processing or preprocessing and lacks deep integration with real-time detection architectures.

Despite this progress, existing crop disease detectors still face three fundamental limitations that constrain practical deployment. First, models often perform well on specific datasets but suffer performance drops when generalized to new environments, crops, or disease types. Second, agricultural applications require high inference speed for high-resolution image streams, yet many accurate models are computationally expensive. Third, lightweight models may meet speed requirements but frequently sacrifice sensitivity to small or early-stage disease lesions. These limitations motivate the development of a detector that jointly improves accuracy, generalization, localization precision, and real-time efficiency.

The objective of this study is to develop and validate a real-time crop disease detection transformer, termed AD-DETR, that can accurately localize disease symptoms in complex agricultural scenes while remaining lightweight enough for edge deployment. The working hypotheses are as follows: (H1) adaptive multi-scale alignment improves the representation of lesions with different sizes and reduces background interference; (H2) spatial–spectral fusion improves disease-feature discriminability by combining local spatial cues with global texture information in the frequency domain; (H3) an improved IoU-based loss function improves bounding-box regression and class-imbalance handling; and (H4) the integration of these components provides a better accuracy–efficiency balance than mainstream CNN- and transformer-based detectors.

Accordingly, the specific objectives of this study are:

1. To design the Multi-Scale Align Network (MSANet) with an Adapt Fusion Align block for adaptive alignment of multi-scale crop disease features;
2. To develop the Spatial–Spectral Attentive Feature Fusion module that combines spatial attention and Fourier-based frequency-domain processing for robust feature representation;
3. To introduce the IPIoUv2 loss function for improved bounding-box regression and more stable localization of irregular disease symptoms; and
4. To evaluate AD-DETR on a large Crop Disease dataset and the PlantDoc dataset, comparing it with representative two-stage, single-stage, YOLO-series, and RT-DETR-based detectors in terms of accuracy, efficiency, and generalization.

## 2. Method

### 2.1. Overall Architecture

**Figure 1:**
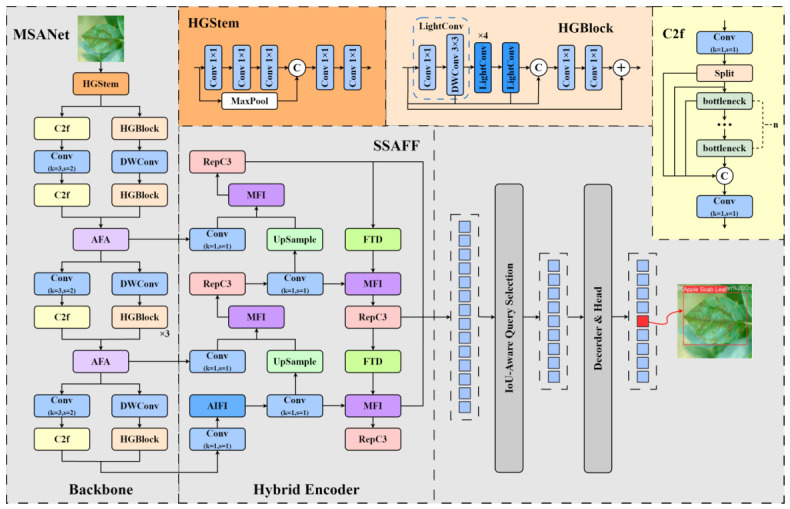
Architecture of AD-DETR for crop disease detection. MSANet = Multi-Scale Align Network; AFA = Adapt Fusion Align; SSAFF = Spatial–Spectral Attentive Feature Fusion; MFI = Multi-scale Focus Integration; FTD = Fourier Transform Downsampling.

The proposed AD-DETR in this paper is a deep learning model particularly designed for detecting crop illness. Its overall architecture is founded upon an enhanced real-time detection transformer framework, and it has been comprehensively optimized for the specific requirements of agricultural scenarios. Seen from Figure 1, the network utilizes an encoder–decoder structure, primarily composed of three core components: the Multi-Scale Align Network (MSANet) backbone network, the Spatial–Spectral Attentive Feature Fusion (SSAFF) model, and an improved detection head. Such a design enables the model to significantly enhance detection precision for multiple categories of crop diseases while maintaining real-time performance.

This paper first designed the Adapt Fusion Align (AFA) block and, based on this block, developed a feature-extraction network named MSANet. This module adaptively aligns multi-scale features to tackle challenges of scale variation and background interference of pest and disease targets in images. Secondly, we innovatively designed the SSAFF model in the encoder part. The innovation of this module lies in its simultaneous processing of spatial- and frequency-domain information. It achieves attention-weighted feature fusion through the Multi-scale Focus Integration (MFI) block, while utilizing the Fourier Transform Downsampling (FTD) block to extract frequency-domain features. This multi-domain fusion strategy allows the model to take full advantage of comprehensive information in illness images, significantly enhancing the discriminative power of the features. Finally, tailored to the characteristics of the disease detection task, we designed the Inner-Powerful-IoUv2 (IPIoUv2) loss function, which significantly improves bounding-box regression accuracy through its internal perception mechanism.

To clarify the uniqueness of AFA, we further compare it with the Squeeze-and-Excitation (SE) mechanism and BiFPN-style weighted fusion. SE recalibrates channels by using global pooled statistics from a single feature map and therefore focuses mainly on channel dependency modeling [27]. BiFPN learns scalar or normalized weights for bidirectional pyramid fusion and is designed for repeated top–down and bottom–up multi-scale aggregation [28]. In contrast, AFA first projects heterogeneous features into a shared channel space, learns spatially varying and channel-aware gates from the concatenated cross-scale features, and then combines these gates with learnable branch-level coefficients. Thus, AFA is not only a channel attention block or a feature-pyramid weighting rule; it is a feature-alignment and fusion unit specifically designed to reduce cross-scale misalignment in crop disease detection. The conceptual differences among AFA, SE, and BiFPN are summarized in Table 1.

### 2.2. Multi-Scale Align Network

Traditional CNNs frequently struggle to efficiently catch multi-scale features, which directly limits the model’s overall performance. To address such an issue, we introduced two modules in MSANet: the HGStem [29] module for low-level feature extraction and C2f module [30] for high-level feature fusion. Both modules dramatically enhance the representational capacity of the network, and their specific structures are illustrated in the Figure 1. To further improve calculational efficiency, we integrated a Depthwise Separable Convolution (DWConv) [31]. DWConv decomposes standard convolution into depthwise and pointwise operations, substantially lowering calculational costs at the same time as maintaining the capability of extracting rich multi-scale features.

**Figure 2:**
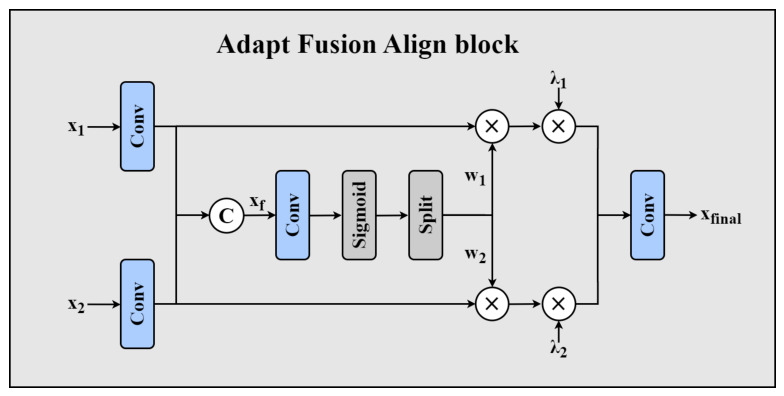
Structure of the AFA block used in MSANet. The two input feature maps are first channel-aligned by 1 × 1 convolution, then fused through the Adapt Align Weight (AAW) mechanism. AFA = Adapt Fusion Align; AAW = Adapt Align Weight.

The AFA block is the core innovative component of MSANet, designed to reduce feature-alignment deviations within the backbone network. Since feature maps often differ in terms of channel count, stride, and receptive field, simple concatenation or weighted averaging can degrade fusion quality. The AFA module solves such an issue by performing feature alignment, adaptive weighting, and channel-wise modulation, thereby achieving more accurate fusion and consequently enhancing the object detection network’s performance. The AFA module’s working principle refers to Figure 2.

In the AFA structure, input features $X=\{x_{1},x_{2}\}$ first undergo a 1×1 convolution for channel alignment, ensuring they are fused within the same feature space. Since $x_{1}$ and $x_{2}$ may originate from different backbone networks, their channel dimensions might not match. Therefore, the following transformations are applied:

(1) $${\hat{x}}_{1}=W_{1}^{\left(1\times 1\right)}⊗x_{1},\;{\hat{x}}_{2}=W_{2}^{\left(1\times 1\right)}⊗x_{2}$$

Here, ⊗ represents the convolution operation; $W_{1}^{\left(1\times 1\right)}$ and $W_{2}^{\left(1\times 1\right)}$ are the 1×1 convolutional weights used for channel transformation, aligning the channel dimensions of $x_{1}$ and $x_{2}$ to lay the foundation for the following fusion.

Feature-aligned ${\hat{x}}_{1}$ and ${\hat{x}}_{2}$ are concatenated post channel alignment. To enhance the adaptability of the fusion, an Adapt Align Weight (AAW) mechanism is introduced. Specifically, a 3×3 convolution is applied to features concatenated to catch high-level fusion information:

(2) $$x_{\mathrm{concat}}=\mathrm{Concat}\left({\hat{x}}_{1},{\hat{x}}_{2}\right),\;x_{f}={\mathrm{Conv}}_{3\times 3}\left(x_{\mathrm{concat}}\right)$$

where ${\mathrm{Conv}}_{3\times 3}$ denotes the 3×3 convolutional layer used to extract fusion information from concatenated features.

Subsequently, the weights are normalized to the interval (0,1) using the sigmoid function σ(·), endowing adaptive weighting of features from varied sources:

(3) $$W_{\mathrm{align}}=\sigma \left(x_{f}\right)$$

The tensor $W_{\mathrm{align}}$ is split along the channel dimension into two independent dynamic weight tensors $w_{1}$ and $w_{2}$:

(4) $$w_{1},w_{2}=\mathrm{Split}\left(W_{\mathrm{align}}\right)$$

Finally, through reasonable weight allocation, AFA dynamically adjusts each input feature’s contribution during the fusion process, achieving smoother and more coordinated feature fusion:

(5) $$x_{\mathrm{fused}}=w_{1}⊙{\hat{x}}_{1}+w_{2}⊙{\hat{x}}_{2}$$

where ⊙ denotes element-wise multiplication, guaranteeing that valid information from varied sources is fused in a more appropriate manner.

But solely relying upon dynamic weights might cause certain feature paths tp be excessively suppressed. To address this, learnable channel weights $\lambda_{1}$ and $\lambda_{2}$ are further introduced to optimize the final fusion ratio:

(6) $$x_{\mathrm{final}}=\lambda_{1}\cdot \left(w_{1}⊙{\hat{x}}_{1}\right)+\lambda_{2}\cdot \left(w_{2}⊙{\hat{x}}_{2}\right)$$

Here, $\lambda_{1}$ and $\lambda_{2}$ are trainable parameters, initialized to 0.5 and automatically optimized during training, allowing the model to learn optimal feature fusion ratio. To prevent gradient explosion or numerical instability during training, the AFA block imposes constraints as shown below on channel weights:

(7) $$\lambda_{1}+\lambda_{2}=1,\;\lambda_{1},\lambda_{2}\in \left[0,1\right]$$

This constraint ensures that the channel weights remain within a steady range, guaranteeing model consistency and generalization capability. Lastly, 1×1 convolution is applied to match features for downstream detection tasks.

### 2.3. Spatial–Spectral Attentive Feature Fusion

Traditional feature fusion methods exhibit significant limitations in crop disease detection: They fail to effectively handle semantic inconsistencies among multi-scale features, leading to inaccurate feature alignment and information loss. Moreover, these methods lack the utilization of frequency-domain information, overlooking the texture and periodic patterns in disease images, while their computational inefficiency makes it difficult to meet real-time detection demands.

To address such issues, this paper designed an SSAFF model. The MFI block applies a multi-scale attention mechanism to dynamically adjust feature weights and prioritize salient disease regions. The FTD block leverages the Fourier transform to map spatial features into the frequency domain for processing, enabling the convolution operation to transcend the limitations of local receptive fields and directly capture global contextual information in images. The entire architecture significantly enhances robustness and preciseness of feature fusion at the same time as keeping real-time performance, providing an efficient solution for crop disease detection.

The MFI block is a core component of Spatial–Spectral Attentive Feature Fusion. Its core innovation lies in integrating local and global attention branches, along with a hierarchical feature processing path to achieve refined fusion of input features. Such a design maintains spatial details of input features and enhances the semantic representation of global contexts via an adaptive weighting mechanism.

**Figure 3:**
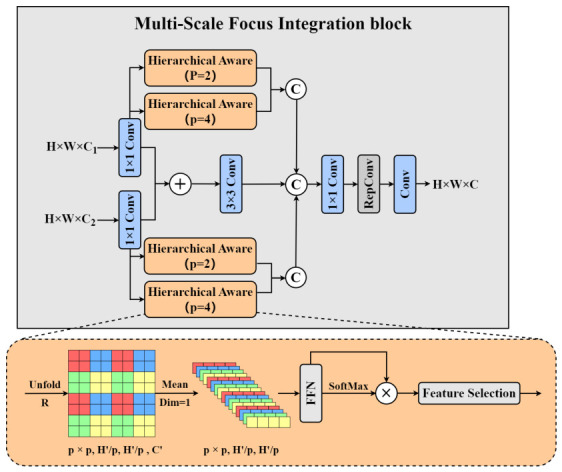
Detailed structure of the MFI and HA blocks in the SSAFF module. MFI = Multi-scale Focus Integration; HA = Hierarchical Aware; SSAFF = Spatial–Spectral Attentive Feature Fusion; *p* is the patch-size parameter used in the HA block, with p=4 and p=2 representing global and local branches, respectively.

Observed from Figure 3, the MFI block workflow consists of the following stages: First, the input features experience a 1×1 convolution for dimensionality reduction. This step is designed to unify the dimensionality of the input features and reduce the computational complexity of subsequent operations. Next, a 3×3 convolution is applied to the dual input features for preliminary fusion, producing baseline features. This provides a stable foundation for subsequent attention-based processing.

Subsequently, these features are processed through the Hierarchical Aware (HA) block. The input feature map is first divided into non-overlapping patches. Let the input feature tensor be $F^{\prime }\in R^{H^{\prime }\times W^{\prime }\times C^{\prime }}$. Through a spatial operation such as unfolding, it is partitioned into patches of size p×p, generating a set of patches $P=\{P_{1},P_{2},\ldots ,P_{N}\}$, where $N=\frac{H^{\prime }\times W^{\prime }}{p\times p}$. This step avoids the information loss associated with uniform downsampling. After simplifying each patch $P_{i}$, it is converted into a patch-level feature vector $t_{i}\in R^{d}$ (where d=p×p).

Task-oriented weighting is then introduced: by comparing the similarity between $t_{i}$ and a task-embedding vector $\xi \in R^{C^{\prime }}$, a weight is computed. A linear transformation $P\in R^{C^{\prime }\times C^{\prime }}$ is applied for channel selection, formulated as:

(8) $${\hat{t}}_{i}=P\cdot \mathrm{sim}\left(t_{i},\xi \right)\cdot t_{i}$$

Here, $\mathrm{sim}(t_{i},\xi )$ is a cosine similarity function, outputting a value in the range [0,1] to measure the relevance of the patch to the task. Patches with high weights are enhanced, while those with low weights are suppressed, achieving adaptive selection.

The weighted patches ${\hat{t}}_{i}$ are then reassembled into a complete feature map via a feature recombination operation. Specifically, all patches are recombined and interpolated back to the original spatial dimensions, producing the enhanced feature $F_{\mathrm{out}}\in R^{H^{\prime }\times W^{\prime }\times C^{\prime }}$, expressed as:

(9) $$F_{\mathrm{out}}=\mathrm{Reshape}\left(\mathrm{Interpolate}\left(\overset{N}{\underset{i=1}{⋃}}{\hat{t}}_{i}\right)\right)$$

Finally, the features processed by the hierarchical perception branch are integrated with the baseline features. The fusion process consists of a 1×1 convolution, a reparameterized 3×3 convolution, and another 1×1 convolution. The use of reparameterized convolution significantly improves parameter efficiency and effectively reorganizes features from multiple branches, ensuring high inference efficiency.

In the feature fusion network of AD-DETR, the FTD block acts as a key component of the SSAFF model, undertaking the critical downsampling function. Unlike traditional downsampling methods based on pooling or strided convolutions, the FTD block innovatively achieves feature reduction in the frequency domain. By combining spectral filtering and resolution adjustment, it reduces feature map resolution while better preserving frequency-domain feature information.

The downsampling process of the FTD block is implemented through frequency-domain operations. Its core idea is to leverage the frequency truncation property of the Fourier transform to achieve resolution reduction. The core mathematical foundation is the convolution theorem: convolution in the spatial domain is equal to element-wise multiplication in the frequency domain. For an N×N input image x(n,m) of size, its discrete Fourier transform (DFT) is defined as:

(10) $$X\left(k_{1},k_{2}\right)=\sum_{n=0}^{N-1}\sum_{m=0}^{N-1}\frac{x(n,m)}{N^{2}}\exp\left[\frac{-2\pi j}{N}\left(nk_{1}+mk_{2}\right)\right]$$

The convolution operation in the frequency domain is expressed as:

(11) $$Y\left(k_{1},k_{2}\right)=X\left(k_{1},k_{2}\right)⊙W\left(k_{1},k_{2}\right)$$

where $W(k_{1},k_{2})$ is a learnable convolution kernel in the frequency domain. The result in the spatial domain can be recovered via the inverse DFT:

(12) IDFT{Y}=x∗w

This mathematical equivalence enables the FTD block to achieve receptive field coverage ranging from local (1×1) to global (N×N).

**Figure 4:**
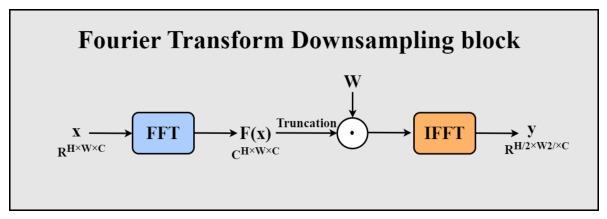
Structure of the FTD block used for frequency-domain downsampling. FTD = Fourier Transform Downsampling; FFT = Fast Fourier Transform; IFFT = Inverse Fast Fourier Transform.

The illustration of FCB is shown in Figure 4. Specifically, given $x\in R^{H\times W\times C}$ input feature map, spatial features are first transformed into the frequency domain via the Fast Fourier Transform (FFT):

(13) X=F(x)

in which F means 2D FFT operation. Since the input consists of real-valued features, the output spectrum displays conjugate symmetry, meaning only half of the frequency-domain data needs to be processed for a complete representation. Then, frequency-domain modulation is applied using a learnable frequency-domain filter $W\in C^{H\times W\times C}$, simultaneously performing frequency truncation:

(14) $$Y=W⊙X_{\mathrm{truncated}}$$

where $X_{\mathrm{truncated}}$ represents the truncated spectrum (retaining low-frequency components), and ⊙ represents element-wise complex multiplication. The learnable filter enables our model to adaptively choose frequency components most important for downsampling. Finally, the result is transformed back to the spatial domain via Inverse FFT (IFFT), naturally achieving resolution reduction:

(15) $$y=F^{-1}\left(Y\right)$$

After the inverse transform, the output feature map y has dimensions $\frac{H}{2}\times \frac{W}{2}\times C$, completing 2× downsampling.

The advantages of this frequency-domain downsampling method are as follows: First, by preserving low-frequency components, it naturally achieves anti-aliasing, avoiding the spectral aliasing issues common in spatial-domain downsampling. Second, the learnable filter can optimize frequency selection for the specific task, enhancing the discriminative power of the downsampled features. Lastly, frequency-domain operations provide a global receptive field, ensuring that long-range dependencies are not lost during the downsampling process.

### 2.4. Inner-Powerful-IoUv2

In crop disease detection tasks, bounding-box regression precision directly affects the model’s localization capability. Traditional IoU loss functions suffer from problems like slow convergence, geometric misalignment, along with limited generalization when handling targets. To address these issues, this paper proposes IPIoUv2, which integrates the geometric alignment penalty mechanism from PowerfulIoUv2 (PIoUv2) [32] and the scale scaling strategy of InnerIoU [33]. By dynamically adjusting the loss weights and adapting to scale variations, our method dramatically enhances bounding-box regression precision, convergence speed, and robustness. The geometric penalty term *P* added by PIoU is calculated directly based on the target bounding box width and height, guiding the anchor box to regress more directly toward the target center and avoiding unnecessary expansion. Its core calculation formula is:

(16) $$P=\frac{1}{4}\left(\frac{d_{w1}}{w^{gt}}+\frac{d_{w2}}{w^{gt}}+\frac{d_{h1}}{h^{gt}}+\frac{d_{h2}}{h^{gt}}\right)$$

(17) $$L_{PIoU}=L_{IoU}+1-e^{-p^{2}},0\leq L_{PIoU}\leq 2$$

where $d_{w1}$, $d_{w2}$, $d_{h1}$, and $d_{h2}$ measure boundary distance disparities between target and predicted boxes in horizontal and vertical directions. $w^{gt}$ and $h^{gt}$ refer to ground-truth box height and width, respectively. Subsequently, based on PIoU, a non-monotonic focal mechanism was introduced to dynamically adjust loss weight, resulting in a new loss function termed PIoUv2. Its calculation formula is defined as follows:

(18) $$q=e^{-p},0<q\leq 1$$

(19) $$u\left(x\right)=3x\cdot e^{-x^{2}}$$

(20) $$L_{PIoUv2}=u\left(\lambda q\right)\cdot L_{PIoU}=3\cdot \left(\lambda q\right)\cdot e^{-{\left(\lambda q\right)}^{2}}\cdot L_{PIoU}$$

among which, λ denotes a hyperparameter controlling penalty strength. Through experiments, we finally set λ=1.3.

The motivation behind Inner-IoU differs from that of PIoU. It primarily addresses the insufficient generalization capability and slow convergence of the traditional IoU loss in disease detection. Its core concept involves introducing a proportionally scaled “auxiliary bounding box” for loss calculation, thereby dynamically adjusting difficulty and focus of regression. The specific calculation formula is as follows:

(21) $$b_{l}^{gt}=x_{c}^{gt}-\frac{w^{gt}*\gamma }{2},b_{r}^{gt}=x_{c}^{gt}+\frac{w^{gt}*\gamma }{2}$$

(22) $$b_{t}^{gt}=y_{c}^{gt}-\frac{h_{gt}*\gamma }{2},b_{b}^{gt}=y_{c}^{gt}+\frac{h_{gt}*\gamma }{2}$$

(23) $$b_{l}=x_{c}-\frac{w*\gamma }{2},b_{r}=x_{c}+\frac{w*\gamma }{2}$$

(24) $$b_{t}=y_{c}-\frac{h*\gamma }{2},b_{b}=y_{c}+\frac{h*\gamma }{2}$$

(25) $$\begin{array}{cc} inter= & (min\left(b_{r}^{gt},b_{r}\right)-max\left(b_{l}^{gt},b_{l}\right))*\\ & \left(min\left(b_{b}^{gt},b_{b}\right)-max\left(b_{t}^{gt},b_{t}\right)\right) \end{array}$$

(26) $$union=\left(w^{gt}*h_{gt}\right)*\gamma^{2}+\left(w*h\right)*\gamma^{2}-inter$$

(27) $$IoU_{inner}=\frac{inter}{union}$$

where $\left(x_{c}^{gt},\;y_{c}^{gt}\right)$ and $\left(x_{c},\;y_{c}\right)$ are the mean ground-truth box and the predicted box center coordinates, respectively. $w^{gt}$, $h^{gt}$, *w*, and *h* represent the ground-truth box and predicted box width and height, respectively. γ means a scale factor, which is set to γ=0.7 here. This setting imposes stricter regression criteria and is capable of accelerating quality samples’ convergence. Therefore, the formula for IPIoUv2 can be expressed as:

(28) $$L_{IPIoUv2}=3\cdot \left(\lambda q\right)\cdot e^{-{\left(\lambda q\right)}^{2}}\cdot \left(2-e^{-p^{2}}-IoU_{inner}\right)$$

## 3. Experiments

### 3.1. Datasets

This study uses two datasets. The first is a self-constructed Crop Disease dataset, and the second is the public PlantDoc benchmark dataset [18]. The Crop Disease dataset contains 37,714 high-quality images covering 41 fine-grained categories. It includes healthy states and common diseases such as early blight, late blight, powdery mildew, bacterial spot, and leaf blight for more than ten important crops, including corn, tomato, grape, strawberry, bell pepper, peach, cherry, potato, apple, citrus, squash, rice, and cassava. Among them, 4850 images are from the Academy of Agricultural Sciences, 7542 images are from the Institute of Intelligent Machinery of the Chinese Academy of Sciences, 10,693 images are from commercial datasets, and 14,629 images are from mainstream search engines. Domain experts used LabelImg to annotate lesion areas and their corresponding categories. After annotation, files were saved in TXT format. The dataset was split into training, test, and validation subsets using a 7:1:2 ratio.

To make the dataset composition clearer, the number of diseased and healthy images used in the experiments is reported in Table 2. In this study, an image is counted as diseased if it contains at least one disease label; otherwise, it is counted as healthy. The self-constructed Crop Disease dataset contains 33,286 diseased images and 4428 healthy images. The PlantDoc object-detection version used for external validation contains 2569 images from 13 plant species and 30 categories, including both diseased and healthy leaf categories, and has 8851 object annotations. PlantDoc was used only for testing cross-dataset generalization and is cited here according to its original dataset paper [18].

The distribution in Table 3 confirms that class imbalance exists, mainly because healthy samples and several visually similar disease categories are less frequent than common diseases such as tomato late blight, corn leaf blight, and tomato early blight. To reduce the influence of this imbalance, the train/validation/test split was performed in a stratified manner, rare categories were augmented using random scaling, flipping, color jittering, and mosaic-style composition, and the IPIoUv2 loss was introduced to improve localization stability for difficult and minority lesion samples.

**Figure 5:**
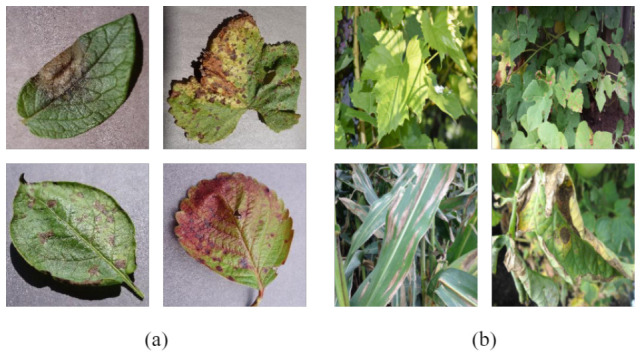
Representative disease images from the datasets. (**a**) shows examples from the Crop Disease dataset, including soybean bacterial blight, strawberry leaf scorch, rice leaf blight, and leaf-spot symptoms. (**b**) shows examples from the PlantDoc dataset, including apple rust, tomato early blight, tomato bacterial spot, and grape leaf disease. Each panel illustrates variation in lesion size, color, texture, and background complexity.

Representative images from both datasets are shown in Figure 5.

### 3.2. Experimental Setup and Evaluation Metrics

The computational infrastructure underpinning this research comprises an NVIDIA GeForce RTX 3090 GPU, collaborating with an Intel Xeon E5-2630 v3 processor (2.40 GHz), housed within a Windows 11 64-bit operational milieu. Model implementation harnesses PyTorch 1.13.1, synergized with Python 3.8, and turbocharged by CUDA 12.1 and cuDNN 8.9.2. An exhaustive enumeration of training configurations is provided in Table 4.

To ensure a fair comparison, all subsequent baseline detectors were trained and evaluated under a unified protocol. The same training/validation/test splits, input size, batch size, number of epochs, image normalization, and data augmentation pipeline were used whenever supported by the official implementation. For two-stage detectors, anchor and region-proposal settings followed the default configuration recommended by the corresponding framework, but the image size and training schedule were kept consistent with the other models. The unified training and evaluation protocol is summarized in Table 5.

To exhaustively appraise the performance of the presented AD-DETR framework and juxtapose it against other avant-garde object identification models, we leverage multiple pivotal metrics quantifying detection precision, computational efficacy, and inference velocity.

Because this study addresses multi-class object detection rather than binary classification, precision and recall are calculated for each class and then averaged across all classes. For class *c*, a detection is counted as a true positive only when its predicted label is correct and its IoU with the corresponding ground-truth box is at least 0.5:

(29) $$P_{c}=\frac{TP_{c}}{TP_{c}+FP_{c}},\;R_{c}=\frac{TP_{c}}{TP_{c}+FN_{c}}.$$

The macro-averaged precision and recall reported in this study are:

(30) $$P=\frac{1}{N_{c}}\sum_{c=1}^{N_{c}}P_{c},\;R=\frac{1}{N_{c}}\sum_{c=1}^{N_{c}}R_{c},$$

where $N_{c}$ is the number of categories, and $TP_{c}$, $FP_{c}$, and $FN_{c}$ denote class-specific true positives, false positives, and false negatives. This formulation better reflects performance across all crop and disease categories, including minority classes.

Mean Average Accuracy at IoU=0.5 (mAP@50): A pervasive metric in object detection, mAP@50 encapsulates the precision–recall curve spanning all object groupings. It entails computing the Average Precision (AP) for each class at an Intersection over Union (IoU) threshold of 0.5, followed by averaging these AP values across all categories. The IoU metric assesses overlap between a forecasted bounding box ($B_{pred}$) and its associated ground-truth bounding box ($B_{gt}$):

(31) $$IoU=\frac{Area(B_{pred}\cap B_{gt})}{Area(B_{pred}\cup B_{gt})}\;$$

The mAP@50 is defined as:

(32) $$mAP@50=\frac{\sum_{c=1}^{N_{c}}AP_{c}}{N_{c}}\left(IoU=0.5\right)$$

$N_{c}$ symbolizes the count of object categories, while $AP_{c}$ i embodies Average Accuracy for category c, computed as the area beneath the accuracy-recall curve specific to that category at IoU=0.5.

Mean Average Accuracy over IoU thresholds 0.5–0.95 (mAP@50–95): Such a metric furnishes a more stringent assessment via averaging mAP computed across a spectrum of IoU thresholds, ranging from 0.5 to 0.95 in 0.05 increments. It rigorously appraises the model’s localization precision across diverse strictness levels.

Parameters (Params): Aggregate sum of trainable parameters within model, quantified in millions (M). Such a metric is critical because it directly impacts the model’s memory footprint and storage requirements.

Floating Point Operations (FLOPs): The computational intricacy of the model, enumerated in Giga FLOPs (GFLOPs). It approximates the requisite floating-point arithmetic operations for a solitary forward pass of an input image. Inferior FLOPs typically suggest superior computational efficiency.

Frames per second (FPS): Evaluates the model’s inference velocity, embodying the number of images that the model is capable of processing per second on designated hardware.

## 4. Results

### 4.1. Ablation Experiments

In order to systematically verify the effectiveness of each core component in the AD-DETR model, we conducted comprehensive ablation experiments. As shown in Table 6, by gradually introducing MSANet, SSAFF and IPIoUv2 modules, the contribution of each component to the model performance and its interaction mechanism were deeply analyzed.

Experimental results show that when MSANet is used alone, the number of model parameters is significantly reduced from the baseline of 19.9 M to 12.3 M, the calculation amount is reduced from 57.0 GFLOPs to 34.4 GFLOPs, and the mAP@50 is increased from 84.8% to 85.0%. This optimization is mainly due to the innovative AFA module design in MSANet, which greatly reduces parameter redundancy while maintaining multi-scale feature-extraction capabilities through deep DWConv and AAW mechanisms. The feature-alignment strategy adopted by the AFA module effectively solves the dimension mismatch problem of traditional convolutional neural networks in cross-scale feature fusion, laying an efficient foundation for subsequent processing.

It is worth noting that when the SSAFF module is introduced alone, the parameter amount increases to 22.3 M and the calculation amount reaches 70.1 GFLOPs, but the mAP@50 is significantly increased to 87.8%. The parameter growth is mainly due to the introduction of frequency-domain processing components in SSAFF. In particular, the FTD block needs to learn complex frequency-domain filters, and the multi-scale attention mechanism in the MFI block increases the computational overhead. However, when SSAFF is combined with MSANet, a significant synergistic effect is produced: the number of parameters is reduced to 16.4 M, which is lower than the baseline model, while mAP@50 is further improved to 89.0%. This “reduction and efficiency increase” phenomenon stems from the deep integration of the two modules—the refined feature alignment provided by MSANet creates a more efficient processing environment for SSAFF, allowing frequency-domain operations to be performed in the compressed feature space, greatly reducing the dimensionality requirements of the frequency-domain filters in the FTD block.

The separate introduction of the IPIoUv2 loss function increases mAP@50 to 86.4%. More importantly, when combined with the complete architecture, it increases mAP@50 to 90.2% while maintaining parameter efficiency. The final AD-DETR model achieved a 5.4% mAP@50 improvement with a 17.2% reduction in parameters, which verifies the synergistic advantages generated through structural optimization and functional complementation between components. This design embodies the concept of “fine preprocessing + intelligent fusion” and provides the optimal accuracy–efficiency balance for real-time disease detection in agricultural scenarios.

### 4.2. Comparison with Representative IoU-Based Losses

To further demonstrate the necessity of IPIoUv2, we replaced only the bounding-box regression loss while keeping the AD-DETR architecture, dataset split, training schedule, and augmentation pipeline unchanged. As shown in Table 7, IPIoUv2 achieved the highest mAP@50 and mAP@50–95. Compared with CIoU and DIoU, IPIoUv2 improved localization by combining geometric penalty and scale-adaptive auxiliary boxes. Compared with WIoU and PIoUv2, it showed better recall, suggesting that the inner-box strategy helps the model learn from small and irregular disease regions.

### 4.3. Comparative Analysis

To evaluate the performance of AD-DETR in crop disease detection in an all-round manner, comparative experiments against cutting-edge object detection architectures, like Faster R-CNN [40], SSD [41], RetinaNet [42], YOLOv8m [43], YOLOv10m [44], YOLOv12m [45], and original RT-DETR variants (r18, r34, r50), were carried out. Experimental outcomes in Table 8 display the superior performance of the proposed approach.

As shown in Table 8, traditional two-stage detectors, including Faster R-CNN, exhibit limited performance with 42.0% accuracy, 35.3% recall, and 36.6% mAP@50, indicating their inadequacy for complex crop disease detection tasks. SSD shows improved precision but suffers from low recall, suggesting significant missed detections in agricultural scenarios.

Among single-stage detectors, RetinaNet achieves competitive results with 84.3% precision and 77.4% recall, while YOLOv8m demonstrates strong recall performance but relatively lower precision. Notably, YOLOv10m achieves the highest precision among all compared methods but shows a noticeable recall drop, highlighting the challenge of balancing precision and recall in disease detection.

The baseline RT-DETR models show consistent performance, with RT-DETR-r50 achieving 89.9% precision and 89.7% recall. However, our proposed AD-DETR outperforms all comparative methods in the critical mAP@50 metric, achieving 90.2% while maintaining excellent precision and recall.

More importantly, AD-DETR achieves this superior performance with the most efficient architecture among all compared methods. With only 16.4 M parameters and 47.2 GFLOPs, our model reduces computational complexity by 17.2% compared to RT-DETR-r18 while improving mAP@50 by 5.4%. The inference speed of 230 FPS further demonstrates ADDETR’s real-time capability, making it appropriate for practical agricultural uses requiring fast disease diagnosis.

Superior performance of AD-DETR can be ascribed to its innovative design: MSANet backbone effectively handles scale variations in disease symptoms, the SSAFF module enhances feature representation through spatial–spectral fusion, and the IPIoUv2 loss function optimizes bounding-box regression for irregular disease patterns. These components work synergistically to address unique challenges of crop illness detection, including small lesion sizes, complex backgrounds, and subtle symptom variations.

Such findings verify that AD-DETR realizes an optimal balance between detection precision and calculational efficiency, making it especially applicable for deployment in resource-constrained agricultural conditions where real-time performance and accuracy are equally important.

### 4.4. Generalization Test

To assess the generalization ability of the AD-DETR model in diverse agricultural environments in an all-around manner, our research performed rigorous cross-dataset validation experiments utilizing the PlantDoc dataset as the test platform. This dataset exhibits significant differences from the primary training dataset, encompassing diverse plant species, disease manifestation patterns, and imaging conditions, thereby effectively testing the model’s robustness to domain shift.

Experimental results in Table 9 demonstrate that AD-DETR achieves outstanding performance on the PlantDoc dataset, with key metrics significantly surpassing those of comparative models. Specifically, AD-DETR realizes 96.4% precision, 94.2% recall, and 97.4% mAP@50. Such outcomes not only substantially exceed those of traditional detectors but also outperform current mainstream deep learning methods. Furthermore, AD-DETR exhibits remarkable efficiency, with only 16.4 M parameters, a computational load of 47.2 GFLOPs, and an inference speed of 242 FPS. This high efficiency makes it highly applicable for resource-constrained real-time agricultural applications, like disease monitoring on drones or mobile devices.

In horizontal comparisons, traditional detectors reveal significant limitations. Faster RCNN achieves only 71.8% mAP@50, and its high parameter count and computational load impede real-time deployment. While SSD shows improved precision, its low recall rate indicates severe missed detections in unfamiliar environments. Among contemporary methods, YOLOv10m is competitive in efficiency but shows a significant gap in mAP@50 compared to AD-DETR. Although a variant of RT-DETR achieves 95.9% mAP@50, its parameter count and calculational load are substantially higher than those of AD-DETR, highlighting the latter’s lightweight advantage.

### 4.5. Visualization of Model Predictions

To qualitatively validate the detection performance and attention mechanisms of the AD-DETR model proposed, this section presents visual comparisons using detection results and Grad-CAM visualizations. These analyses provide intuitive insights into the model’s capability to precisely localize disease spots and focus on relevant regions, complementing the quantitative metrics discussed earlier.

**Figure 6:**
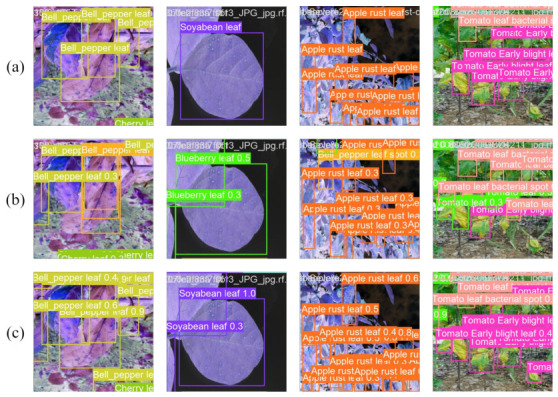
Detection comparison between RT-DETR and AD-DETR on representative crop disease images. (**a**) shows the original images; (**b**) shows RT-DETR results; (**c**) shows AD-DETR results. The sample diseases include bell pepper bacterial spot, soybean leaf disease, apple rust, tomato bacterial spot, and tomato early blight.

Figure 6 illustrates a comparative visualization of detection outcomes between the original RTDETR model and our AD-DETR on representative crop disease images. The results clearly demonstrate that RT-DETR suffers from significant issues, including false positives and missed detections. In contrast, AD-DETR achieves precise detection without such errors, accurately bounding all disease spots while minimizing background interference. This visual evidence confirms the effectiveness of AD-DETR in handling the diverse manifestations of diseases in complex agricultural scenarios.

**Figure 7:**
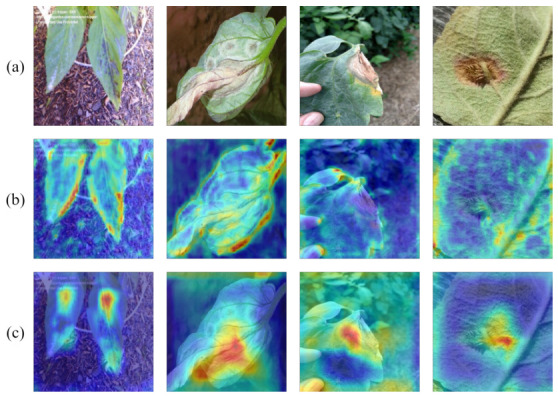
Grad-CAM visualization for representative crop disease samples. (**a**) shows original images; (**b**) shows RT-DETR attention maps; (**c**) shows AD-DETR attention maps. The examples include leaf blight, early blight-like lesions, bacterial spot, and rust-like symptoms.

Additionally, Figure 7 showcases Grad-CAM visualizations to analyze the spatial focus of the models. The heatmaps generated by RT-DETR exhibit a dispersed attention pattern, often highlighting irrelevant background areas or only partially covering disease regions, which contributes to its suboptimal performance. Conversely, AD-DETR’s heatmaps are highly concentrated on the core disease areas, indicating a more targeted and reliable feature-extraction process. This enhanced focus can be attributed to the SSAFF model, which integrates frequency-domain information to improve feature discriminability. The sharper heatmaps confirm that AD-DETR effectively suppresses noise and prioritizes salient disease features, leading to superior localization accuracy.

**Figure 8:**
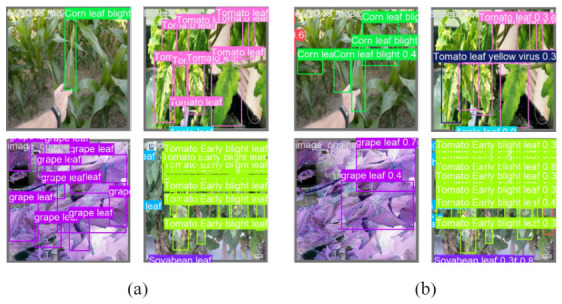
False-positive and false-negative cases of AD-DETR under challenging conditions. (**a**) shows original images; (**b**) shows AD-DETR detection results. Disease names visible in the samples include corn leaf blight, tomato yellow leaf curl virus, grape leaf disease, and tomato early blight. These cases illustrate errors caused by low contrast, occlusion, background similarity, and overlapping leaves.

As shown in Figure 8, AD-DETR still makes errors when disease symptoms are similar to healthy leaf veins, under low contrast, or in the presence of severe occlusion. These cases reveal the need for further improvement in difficult field environments.

### 4.6. Confusion Matrix and Error Analysis

**Figure 9:**
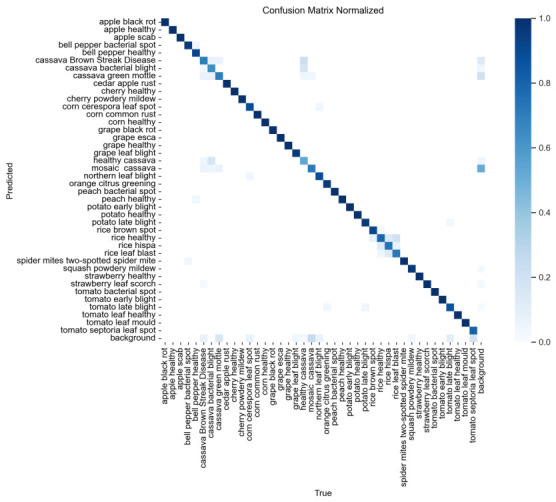
Normalized confusion matrix for representative high-frequency crop disease classes. The matrix is row-normalized; darker diagonal cells indicate correct detections, whereas off-diagonal cells indicate misclassification or confusion between visually similar disease categories.

To further analyze the class-wise detection performance of AD-DETR, a normalized confusion matrix was generated for the Crop Disease validation set, as shown in Figure 9. The matrix includes all 41 crop disease/healthy categories and the background category. The horizontal axis represents the true class, while the vertical axis represents the predicted class. Predictions were matched with ground-truth boxes using an IoU threshold of 0.5. Diagonal elements indicate correct detections, whereas off-diagonal elements indicate misclassification or confusion with the background.

As shown in Figure 9, most categories present strong diagonal responses, indicating that AD-DETR correctly detects the majority of crop disease classes. The off-diagonal values are generally weak, suggesting that the proposed model has good discriminative ability across multiple crops and disease types. However, some errors still occur between visually similar categories. For example, several tomato diseases, such as early blight, late blight, bacterial spot, Septoria leaf spot, and leaf mold, may be confused because they share similar necrotic spots, yellowing symptoms, and irregular lesion boundaries. Similar confusion can also be observed among diseases from the same crop, such as grape black rot, grape leaf blight, and grape esca, as well as potato early blight and potato late blight.

In addition, some elongated leaf-lesion diseases, such as corn leaf blight, rice leaf blast, and rice brown spot, may be confused when lesions are small, low-contrast, or distributed along leaf veins. Cassava-related categories also show several off-diagonal responses, mainly because mottling, chlorosis, and weak color transitions make their visual differences less obvious. False positives are mainly caused by healthy veins, shadows, or senescent leaf edges that resemble disease texture, while false negatives usually occur under small-lesion, occlusion, blurred-image, or low-contrast conditions. These findings are consistent with the qualitative error cases shown in Figure 8.

Overall, the confusion matrix confirms that AD-DETR achieves strong class-wise detection performance, with most errors concentrated in visually similar diseases and difficult background conditions. Future work should further improve the recognition of early-stage and low-contrast symptoms by using more balanced samples, hard negative mining, and higher-resolution lesion features.

## 5. Discussion

The objective of this study was to develop and validate a real-time transformer detector for crop disease detection that improves localization accuracy, generalization, and computational efficiency. The results support the proposed hypotheses. AD-DETR achieved 90.2% mAP@50 on the Crop Disease dataset and 97.4% mAP@50 on PlantDoc while retaining 16.4 M parameters, 47.2 GFLOPs, and real-time inference speed. These findings indicate that adaptive multi-scale alignment, spatial–spectral fusion, and improved regression loss jointly improve the accuracy–efficiency balance of crop disease detection.

The first hypothesis, that adaptive multi-scale alignment improves disease-feature representation, is supported by the ablation results. MSANet reduced parameters from 19.9 M to 12.3 M and slightly improved mAP@50 when used alone. More importantly, when MSANet was combined with SSAFF, the model reached 89.0% mAP@50 while keeping parameters at 16.4 M. This result is consistent with prior evidence that multi-scale feature representation is critical for object detection. In crop disease images, lesions vary from small early-stage spots to large necrotic areas. A model that aligns cross-scale features can therefore reduce missed detections caused by scale mismatch and background interference.

The second hypothesis, that spatial–spectral fusion improves disease discrimination, is also supported. SSAFF increased mAP@50 to 87.8% when used alone, despite the additional computational cost. This indicates that frequency-domain cues can provide complementary information to spatial features. Prior plant disease studies have shown that leaf lesions often include subtle texture, color-transition, and edge-distribution patterns. Unlike methods that use frequency-domain operations as preprocessing, SSAFF integrates Fourier-based downsampling into the detection architecture, allowing global texture information to interact with attention-based spatial features. This helps the model concentrate on disease regions, as shown by the sharper Grad-CAM responses.

The third hypothesis, that the improved IoU-based loss enhances localization, is supported by the performance of IPIoUv2. When IPIoUv2 was introduced alone, mAP@50 improved to 86.4%, and the complete model achieved the highest mAP@50 and mAP@50–95. Irregular lesion boundaries and imbalanced class distributions are common in plant disease detection, and conventional bounding-box regression may converge slowly or produce suboptimal localization. By combining geometric penalties and scale-adaptive auxiliary boxes, IPIoUv2 provides stricter localization guidance for high-quality samples while improving stability for difficult targets.

Compared with previous crop disease studies, AD-DETR addresses a different and more deployment-oriented task. Several CNN-based studies have reported high classification accuracy under curated or semi-controlled image conditions. However, classification models often identify the image-level disease category without localizing the lesion area, limiting their utility for severity assessment, targeted treatment, and field monitoring. Object detection studies such as improved Faster R-CNN and YOLO-based detectors can localize disease regions, but they may trade speed for accuracy or show reduced robustness under scale variation. In this study, AD-DETR surpassed Faster R-CNN, SSD, RetinaNet, YOLOv8m, YOLOv10m, YOLOv12m, and RT-DETR variants in mAP@50 on the Crop Disease dataset while maintaining real-time speed. These comparisons suggest that the proposed design better balances lesion localization, computational cost, and inference velocity.

The generalization results on PlantDoc further demonstrate the potential of AD-DETR for real agricultural scenarios. PlantDoc contains heterogeneous images with diverse plant species, disease symptoms, and field conditions. AD-DETR achieved 97.4% mAP@50 on this dataset, outperforming RT-DETR-r50 while requiring fewer parameters and lower computational cost. This finding is particularly important because real-world deployment often involves images collected under variable lighting, crop growth stages, camera distances, and background complexity. The result supports the fourth hypothesis that the integrated architecture improves accuracy and efficiency under domain shift.

The broader implication of this work is that transformer-based detection can be adapted to agricultural edge intelligence when architectural components are designed for field-specific challenges. The lightweight and fast AD-DETR model may support real-time monitoring on unmanned aerial vehicles, mobile phones, field robots, and greenhouse cameras. Accurate lesion localization can assist precision spraying, early warning, yield-loss reduction, and disease-spread monitoring. Because the model produces bounding boxes rather than only class labels, its outputs may also support downstream disease severity estimation, treatment prioritization, and agronomic decision-making.

Nevertheless, the model still has limitations. First, performance may decline under extreme lighting, strong occlusion, or backgrounds that resemble disease texture, as shown in the error cases. Second, although the Crop Disease dataset covers many categories, rare diseases and early-stage symptoms may remain underrepresented. Third, frequency-domain downsampling improves global texture representation but may still lose some high-frequency details that are useful for very small lesions. Fourth, this study focuses on RGB images; environmental variables such as temperature, humidity, and crop growth stage were not incorporated. Future work should expand multimodal data sources, combine visual and environmental sensor information, and evaluate the model in long-term field trials.

Future research will focus on three directions. First, multimodal sensing will be introduced to improve robustness under complex field conditions. Second, adaptive lightweight strategies will be developed through dynamic network structures to reduce computation for edge devices. Third, incremental and continual learning mechanisms will be explored so that AD-DETR can adapt to new disease types without full retraining. These directions will help extend the practical value of AD-DETR in precision agriculture.

## 6. Conclusions

This paper proposes AD-DETR, a real-time detection framework specifically designed for crop disease detection. Through multi-scale feature alignment, spatial–spectral fusion, and bounding-box regression optimization, it effectively addresses key challenges in agricultural computer vision. Experimental validation shows that AD-DETR achieves state-of-the-art performance on multiple datasets while maintaining efficient computational characteristics. Specifically, on the Crop Disease dataset, the model achieves 90.2% mAP@50, which is a 5.4% improvement over the baseline. The parameter count is only 16.4 M, with a computational complexity of 47.2 GFLOPs and an inference speed of 242 FPS. In generalization tests on the PlantDoc dataset, the mAP@50 reaches 97.4%, confirming its practicality in real-world agricultural environments.

Methodologically, the core innovations of AD-DETR include: MSANet, which achieves adaptive alignment of multi-scale features through the AFA block, significantly reducing feature bias and parameter redundancy; the SSAFF module, which integrates spatial attention and frequency-domain processing to enhance the discriminative capability of disease features; and the IPIoUv2 loss function, which combines geometric penalties and scale-adaptive strategies to optimize the precision and convergence speed of bounding-box regression. Ablation experiments validate the independent contributions and synergistic effects of each component, while the overall architecture achieves a balance between accuracy and efficiency while maintaining lightweight characteristics.

Although AD-DETR performs excellently in most scenarios, it still has limitations. Future research can be expanded in three aspects: first, incorporating multimodal data sources and combining environmental sensor information to improve model robustness; second, developing adaptive lightweight strategies to optimize computational efficiency through dynamic network structures; and third, exploring incremental learning mechanisms to enable the model to continuously adapt to new disease types. These directions will promote the broader application of AD-DETR in precision agriculture.

Overall, through methodological innovations and empirical validation, AD-DETR provides an efficient and reliable solution for crop disease detection and is expected to extend to broader areas of plant science and precision agriculture.

## Figures and Tables

**Table 1:** Conceptual comparison among AFA, SE, and BiFPN. AFA is designed for adaptive cross-scale feature alignment, whereas SE mainly recalibrates channel responses and BiFPN learns pyramid-level fusion weights.

Method	Input Condition	Weight Generation	Fusion/Alignment Behavior	Main Difference from AFA
SE	Single feature map	Global pooling and channel excitation	Recalibrates channel responses within one scale	Does not explicitly align two heterogeneous feature maps or model cross-scale spatial offsets
BiFPN	Multi-scale feature pyramid	Learnable normalized scalar weights	Repeated bidirectional pyramid fusion	Uses scale-level weights but does not perform feature-specific channel projection and spatially varying gated alignment
AFA (ours)	Two heterogeneous cross-scale features	Concatenation, 3 × 3 convolution, sigmoid split, and learnable branch coefficients	Channel alignment, spatial-channel gating, and adaptive branch fusion	Jointly performs feature-space alignment and adaptive cross-scale fusion for small and irregular lesions

**Table 2:** Dataset statistics used in this study. The Crop Disease dataset was constructed in this work; PlantDoc is a public benchmark dataset cited from Singh et al. [18]. Images with at least one annotated disease region were counted as diseased images.

Dataset	Source/Citation	Total Images	Crops/Species	Categories	Diseased Images	Healthy Images
Crop Disease	Self-constructed in this study	37,714	>10 crops	41	33,286	4428
PlantDoc	Public benchmark [18]	2569	13 species	30	2118	451

**Table 3:** Image distribution of the 41 categories in the self-constructed Crop Disease dataset. The distribution shows that the dataset contains class imbalance, with healthy categories generally having fewer images than major disease categories.

Category	Images	Ratio (%)	Category	Images	Ratio (%)	Category	Images	Ratio (%)
apple healthy	420	1.11	bell pepper bacterial spot	1111	2.95	cassava bacterial blight	1159	3.07
bell pepper healthy	390	1.03	cherry powdery mildew	1037	2.75	cassava green mottle	1098	2.91
cherry healthy	350	0.93	corn perispore leaf spot	1184	3.14	strawberry leaf scorch	1050	2.78
corn healthy	430	1.14	corncommon rust	1135	3.01	tomato early blight	1196	3.17
grape healthy	400	1.06	northern leaf blight	1111	2.95	tomato late blight	1208	3.20
peach healthy	320	0.85	grape blackrot	1123	2.98	tomato bacterial spot	1184	3.14
potato healthy	380	1.01	grape leaf blight	1086	2.88	pider mites two-spotted spider mite	1135	3.01
rice healthy	440	1.17	grapeesca	1050	2.78	cassava Brown Streak Disease	1074	2.85
cassava healthy	410	1.09	peach bacterial spot	1037	2.75	tomato leaf mould	1062	2.82
strawberry healthy	330	0.88	potato early blight	1111	2.95	tomato septoria leaf spot	1098	2.91
tomato leaf healthy	558	1.48	potato late blight	1123	2.98	quash powdery mildew	1074	2.85
apple black rot	1062	2.82	rice hispa	1172	3.11	orange citrus greening	1147	3.04
cedar apple rust	1147	3.04	rice brown spot	1086	2.88	mosaiccassava	1054	2.79
apple scab	1074	2.85	rice leaf blast	1098	2.91			

**Table 4:** Training hardware and hyperparameter configuration used for AD-DETR experiments.

Parameter	Configuration
Training epochs	200
Batch size	16
Workers	4
Learning rate	0.0001
Optimizer	AdamW (implemented in PyTorch v1.13.1)
Input image size	640 × 640

**Table 5:** Unified training and evaluation protocol for comparison models. All models used the same dataset split, input resolution, and timed inference procedure.

Model Group	Epochs	Input Size	Batch Size	Optimizer/Scheduler	Data Augmentation
Faster R-CNN, SSD, RetinaNet	200	640×640	16	SGD/AdamW with cosine decay	Resize, horizontal flip, color jitter, random crop
YOLOv8m, YOLOv10m, YOLOv12m	200	640×640	16	AdamW with cosine decay	Mosaic, random scale, flip, HSV/color jitter
RT-DETR variants	200	640×640	16	AdamW with cosine decay	Resize, random scale, flip, color jitter
AD-DETR (ours)	200	640×640	16	AdamW with cosine decay	Same as RT-DETR plus class-balanced rare-category augmentation

**Table 6:** Ablation analysis of MSANet, SSAFF, and IPIoUv2 on the Crop Disease dataset. The best results are shown in bold.

MSANet	SSAFF	IPIoUv2	P	R	*mAP*@50	*mAP*@50–95	Params/M	GFlops/G
×	×	×	0.867	0.854	0.848	0.820	19.9	57.0
✓	×	×	0.870	0.853	0.850	0.816	12.3	34.4
×	✓	×	0.893	0.880	0.878	0.844	22.3	70.1
×	×	✓	0.896	0.859	0.864	0.829	19.9	57.0
✓	✓	×	0.903	0.892	0.890	0.859	16.4	47.2
×	✓	✓	**0.910**	**0.899**	0.894	0.861	22.3	70.1
✓	✓	✓	0.898	0.895	**0.902**	**0.868**	**16.4**	**47.2**

**Table 7:** Comparison of representative IoU-based losses on the Crop Disease dataset. Only the regression loss was changed; the network architecture and training protocol were unchanged. The best results are shown in bold.

Regression Loss	P	R	mAP@50	mAP@50–95
GIoU [34]	0.895	0.887	0.893	0.858
DIoU [35]	0.893	0.884	0.890	0.855
CIoU [36]	0.889	0.878	0.884	0.848
EIoU [37]	0.891	0.881	0.887	0.852
SIoU [38]	0.884	0.872	0.879	0.841
WIoU [39]	0.896	0.889	0.895	0.860
PIoUv2	0.897	0.891	0.897	0.862
Inner-IoU	0.894	0.890	0.896	0.861
IPIoUv2 (ours)	**0.898**	**0.895**	**0.902**	**0.868**

**Table 8:** Performance comparison on the Crop Disease dataset. The best results are shown in bold.

Network	P	R	*mAP*@50	Params/M	GFLOPs/G	FPS
Faster RCNN	0.420	0.353	0.366	41.4	134.0	23
SSD	0.657	0.386	0.512	24.8	217.0	84
Retinanet	0.843	0.774	0.793	36.5	130.0	76
YOLOv8m	0.755	0.864	0.829	25.9	79.3	170
YOLOv10m	**0.931**	0.809	0.872	16.6	64.5	222
YOLOv12m	0.753	**0.901**	0.888	20.2	68.1	228
RT-DETR-r18	0.867	0.854	0.848	19.9	56.9	211
RT-DETR-r34	0.870	0.853	0.849	31.1	88.9	147
RT-DETR-r50	0.899	0.897	0.885	41.9	129.6	97
**Ours**	0.898	0.895	**0.902**	**16.4**	**47.2**	**230**

**Table 9:** Cross-dataset performance comparison on the PlantDoc dataset. The best results are shown in bold.

Network	P	R	*mAP*@50	Params/M	GFLOPs/G	FPS
Faster RCNN	0.705	0.672	0.718	41.4	134.0	15
SSD	0.857	0.763	0.862	24.8	217.0	67
Retinanet	0.867	0.828	0.873	36.5	130.0	70
YOLOv8m	0.683	0.701	0.750	25.9	79.3	155
YOLOv10m	0.742	0.849	0.873	16.6	64.5	**269**
YOLOv12m	0.693	0.655	0.749	20.2	68.1	204
RT-DETR-r18	0.861	0.853	0.903	19.9	56.9	201
RT-DETR-r34	0.912	0.873	0.929	31.1	88.9	145
RT-DETR-r50	0.950	0.912	0.959	41.9	129.6	101
**Ours**	**0.964**	**0.942**	**0.974**	**16.4**	**47.2**	242

## Data Availability

Due to the nature of this research, participants in this study did not agree for their data to be shared publicly, so supporting data are not available.
